# Pain management for people with dementia: a cross-setting systematic review and meta-ethnography

**DOI:** 10.1177/20494637221119588

**Published:** 2022-09-16

**Authors:** Toby O Smith, Dawn Lockey, Helen Johnson, Lauren Rice, Jay Heard, Lisa Irving

**Affiliations:** 1 School of Health Sciences, University of East Anglia, Norwich, UK; 2 Nuffield Department of Orthopaedics, Rheumatology and Musculoskeletal Sciences, 6396 University of Oxford, Oxford, UK; 3 Physiotherapy Department, South Tyneside and Sunderland NHS Foundation Trust, 156655 Sunderland Royal Hospital, Sunderland, UK; 4 Physiotherapy Department, Lewisham and Greenwich NHS Trust, Lewisham, 156760 University Hospital Lewisham, London, UK

**Keywords:** Pain, discomfort, cognitive impairment, agitation, care home, qualitative

## Abstract

**Background:**

Pain management for people with dementia is challenging. There is limited understanding on the experiences of pain management from people with dementia, but also from those who support them. This study synthesised the qualitative evidence to explore the perspectives of people with dementia, their family, friends, carers and healthcare professionals to pain management.

**Methods:**

A systematic literature review was undertaken of published and unpublished literature databases (to 01 November 2021). All qualitative research studies reporting the perspectives of people with dementia, their family, friends, carers and healthcare professionals to managing pain were included. Eligible studies were appraised using the Critical Appraisal Skills Programme (CASP) qualitative appraisal tool. A meta-ethnography analysis approach was adopted, with findings assessed against the GRADE-CERQual framework.

**Results:**

Of the 3994 citations screened, 33 studies were eligible. Seven themes were identified from the data. There was moderate evidence from six studies indicating inequity of pain management for people with dementia. There was moderate evidence from 22 studies regarding anxieties on cascading pain information. There was moderate evidence from nine studies that familiarisation of the person with pain, their preferences, routines and behaviours were key factors to better pain management. Consistently, carers and healthcare professionals had a low opinion of the management of pain for people with dementia, with tensions over the ‘best’ treatment options to offer. This was associated with poor training and understanding on how pain ‘should’ be managed.

**Conclusion:**

The findings highlight the challenges faced by people with dementia and pain, and those who support them. Improvements in education for people who support these individuals would be valuable across health and social care pathways. Supporting family members and relatives on pain experiences and treatment options could improve awareness to improve quality of life for people with dementia and pain and those who support them.

## Introduction

Dementia is a major, worldwide health challenge. It has a global prevalence of 45 million people.^1^ Pain is frequently reported in older people. Approximately 20%–50% of older people experience pain.^2^ For these people, pain predominantly, but not exclusively, arises from the musculoskeletal system. Osteoarthritis and pain caused by falls, pressure ulcers, infections and neuropathy are common sources.^3^

The evidence on the epidemiology of pain for people with dementia has been poorly reported.^4^ It has been estimated that between 40% and 80% of people living with dementia in care homes experience significant acute or chronic pain.^5^ This estimated pain prevalence is concordant with older adults without dementia.^6^ There remains conflicting research on pain sensitivity for older people and those with dementia. Whilst some studies indicate a modest decrease in age-related pain sensitivity,^7^ others report reduced pain threshold.^8^ From a treatment perspective, pathological changes such as gliosis and neuronal death, coupled with reduced descending pain inhibitory mechanisms, may decrease the efficacy of pain treatments with advancing age.^9^

Managing pain in people with dementia can be difficult. From an assessment perspective, challenges often relate to communication difficulties. Agitation reported by people with dementia may derive from pain, but equally may result of frustration, hunger, thirst constipation or difficulties communicating other needs. Knowing whether agitated behaviour is pain-related can therefore be difficult. People with dementia may also find it difficult to engage with more participatory interventions such as exercise and medication taking, which are regarded as cornerstone interventions for pain management.^10^ Furthermore, from a pharmacology perspective, comorbidities and the potential risks of increasing confusion and agitation, mean healthcare professionals may feel conflicted on analgesic prescription decisions.^8^ Previous quantitative literature has reported the challenges of under-reporting and under-treating pain in people with dementia living in the community or care facilities.^6^

People with dementia are supported day-to-day by a variety of individuals. These may include family members and friends as informal caregivers, paid carers either in-reaching into homes as domiciliary home support or, if individuals live in care facilities, as carers in residential or nursing homes. Both groups of individuals can be supported by healthcare professionals such as general practitioners, geriatricians and geriatric psychiatrists, rheumatologists and pain consultants, nurses, physiotherapists and occupational therapists. Whilst numerous groups have a vested interest in the health and wellbeing of people with dementia, previous literature has suggested a lack of knowledge, training and uncertainty on how to support these indviduals.^11^ This is a major failing as poor pain management for people with dementia not only reduces their quality of life but also impacts on those who support them where the behaviours and personal needs of the person with dementia and pain may be met more easily when the individual is less agitated, distressed and impacted by pain.

Whilst there has been some qualitative research exploring the perspectives of informal/formal carers and healthcare professionals supporting these people, no studies have attempted to explore interactions in the perspectives of all groups through a meta-ethnography. Only Geddis-Regan et al.^12^ have undertaken a meta-ethnography exploring pain with people with dementia. However, this focused on orofacial pain and with healthcare professionals. The authors expanded this to explore whole-body pain (not just orofacial).^12^ They reported the importance of family members and care teams to assist healthcare professions to determine when and how to act on pain management strategies.^12^ Whilst this provided valuable insights, the search was performed to 2017. The purpose of this study was to update this search and to explore the perspectives of pain management for people with dementia, by people with dementia, their family, friends, carers and healthcare professionals, using a meta-ethnography approach.

## Methods

This systematic review was reported following the Preferred Reporting Items for Systematic Review and Meta-Analyses (PRISMA) guidelines^13^ and the eMERGe reporting guidance.^14^ It was registered in the PROSPERO international prospective register of systematic reviews (CRD42021284840).

### Eligibility criteria

We framed the eligibility criteria using the SPIDER tool.^15^ Through this, studies were included if they met the following:

Sample: Individuals with dementia and pain living at home, in a care institution or in hospital/health service setting and family members and friends, informal and formal caregivers and healthcare professionals who support people living with dementia and pain. We excluded individuals whose cognitive impairment was suggested as temporary (less than 3 months) such as delirium and where pain management was associated with end-of-life care.

Phenomenon of interest: Pain management and supporting people with dementia, living with pain.

Design: Published literature of any research design.

Evaluation: Views or experiences.

Research type: Qualitative and mixed methods peer-reviewed studies.

### Search strategy

The search was undertaken by one reviewer (TS) using published literature databases including Embase, MEDLINE, CINAHL and PubMed to ensure relevant health journals could be identified. Furthermore, the adoption of multiple literature databases may mitigate the risk of omitting potentially eligible qualitative research.^16^ We accessed unpublished or ongoing study data from registries including the WHO International Clinical Trial Registry and ClinicalTrials.gov. The search strategy adopted for Embase is presented as Supplementary File 1. This was adapted for each database. We placed no restriction on the search for date of publication, risk of bias or language of publication. Searches were performed from database inception to 1st November 2021.

To augment the principal search strategy, a forward-citation search was performed for all included studies using the Scopus database. Secondly, a backward-citation search was conducted through a review of all included study reference lists.

### Study identification

Two reviewers (TS and JH) independently reviewed all titles and abstracts from the search results. Full-text papers for all potentially eligible studies were independently reviewed to determine final inclusion. Disagreements between the two reviewers were resolved through discussion.

### Data extraction

We extracted data onto a pre-defined data extraction form. This was developed by the review team to ensure all relevant review data were capture prior to commencing. This was piloted for three initial studies across the data extraction team to promote consistency in the data extracted prior to the full data extraction phase.

Data extracted included country of origin, year of study, number and characteristics of participants including data on age and gender, residential status and location, pain-diagnosis and severity, medical comorbidities, type of dementia, severity of dementia, healthcare professional or formal caregiver characteristics, that is, profession, location, relationship to person with dementia, perspective, attitudes, experiences and views of respondents (patients, family members and friends (informal caregivers), formal caregivers and healthcare professionals) towards pain assessment and/or treatment.

Data extraction was performed by one reviewer (TS) and verified by a second reviewer (LI, DL, HJ or LR). Disagreements between the reviewers were resolved through discussion. If the same study was reported across two or more papers, these were classified as a single study to avoid multiple participant-counting.

### Methodological quality assessment

Each included study was critically appraised using the Critical^17^ Appraisal Skills Programme (CASP) qualitative appraisal tool. This is a 10-item critical appraisal checklist which was specifically designed to assess internal and external validity of qualitative research studies. Each item was graded as a satisfied (yes) or not satisfied (no) assessment.

The critical appraisal was performed by one reviewer (TS) and verified by second (LI, DL, HJ or LR). Disagreements between reviewers were resolved through discussion.

### Data synthesis

We analysed the data using a meta-ethnography approach. This is one of the most frequently used methods for reviewing and synthesising the findings of published qualitative research.^18^ Data from eligible studies were interpreted in-line with a meta-ethnography approach using first-, second- and third-order analysis constructs.^19^ First-order constructs were primary themes reflecting participant’s understandings extracted from the ‘results’ sections of included studies. Second-order constructs were the interpretations of participant’s understandings made by authors, extracted from the ‘discussion’ sections of included studies. Finally, third-order constructs were generated by reviewers (TS, LI, DL, HJ and LR) through discussion and interpretation of the first- and second-order constructs. This is explained below.

First-order themes were grouped into categories independently by reviewers (TS, LI, DL, HJ, and LR). Categories were created based on primary data from the included studies rather than using wider literature or previous scoping searches.^20^ These were tabulated and used to develop a conceptual map. The findings were discussed amongst the review team. This was repeated for all second-order constructs. We labelled where the first- and second-order constructs were derived for each code to explore how the papers and constructs related to one another. Such constant comparative techniques were used to compare how emergent categories related to the primary data/original texts in their similarities (reciprocal analysis) and in their contradictions (refutational analysis). We translated second‐order to third‐order constructs identified in interpretive analysis, checking translations in iterative cyclical processes.^21^ The analysis of these findings was collapsed into interpretive themes to develop a line of argument.^19,20^

Findings were assessed for confidence using the GRADE-CERQual (Confidence in the Evidence from Reviews of Qualitative research) tool.^22^ This was based on four components: (1) methodological limitations, (2) coherence (consistency across primary studies), (3) adequacy of data (the degree of richness and quantity of data supporting the review finding) and (4) relevance. Using these, second-order review findings were graded from ‘high’ ‘moderate’ to ‘low’ and ‘very low’ certainty evidence.

## Results

### Search results

**Figure 1.**
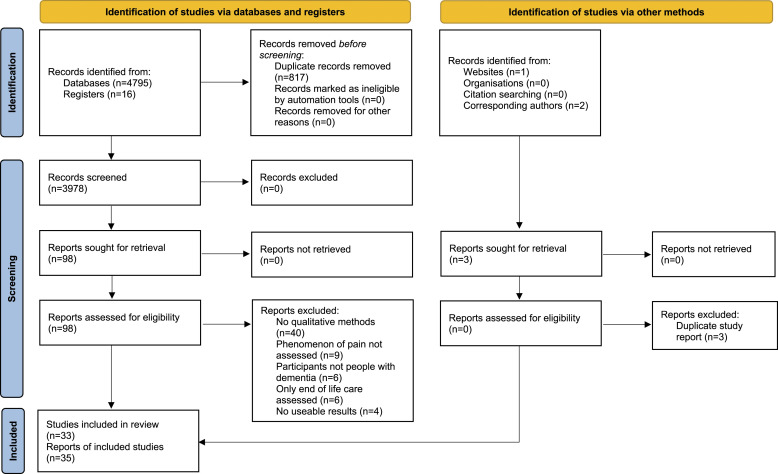
**PRISMA** flow chart summarising the results of the search strategy.

A summary of the search results is presented in Figure 1. In total, 3994 citations were screened for eligibility. Of these, 98 were deemed potentially eligible. On final review, 33 studies (35 publications) were eligible and included in the analysis.

### Characteristics of included studies

The 33 studies originated across Europe, North America, Australasia and Asia. As Table 1 illustrates, most studies originated from the UK^23–33^ (*n* = 10), Australia^34–39^ (*n* = 6), Sweden^40–44^ (*n* = 5), USA^45–49^ (*n* = 5) and Canada^50–52^ (*n* = 3).

**Table 1.** Characteristics of included studies where the participants were people with pain and/or their family members/friends/informal caregivers.

Study	Country origin	Data collection method	Patients				Caregiver			
			N	Gender (M/F)	Mean age (years)	Pain disorder	N	Gender (male/female)	Mean age (years)	Relationship to patient
Barry^30^	UK	Interviews	42	18/24	82.1	N/S	35	8/27	51.8	Child – 23
										Sibling – 12
Bullock^54^	UK	Interviews	8	6/2	73.5	Spinal pain – 3	9	4/5	68	Spouse – 7
						Arthritis – 4				Father – 1
						Tooth pain – 1				Son – 1
Corbett^3^	UK	Focus groups	N/A	N/A	N/A	N/S	12	N/S	N/S	N/S
Lichtner^26^	UK	Observations and interviews	31	11/20	88	Acute hospital admission	4	N/S	N/S	N/S
Liu^53^	China	Interviews	5	1/4	85.6	Arthritis – 4	5	2/3	N/S	Spouse – 3
						LBP – 1				
										Daughter/son – 2
						Fracture – 1				
Malhortra^60^	Singapore	Interviews	N/A	N/A	N/A	N/S	27	21/6	50–69	Spouse – 5
										Daughter/son – 17
										Sibling – 1
										Daughter in law – 3
										Grandchild – 1
Martin^53^	Canada	Focus groups	12	N/S	N/S	N/S	8	N/S	N/S	N/S
Mentes^45^	USA	Interviews	20	7/13	82	N/S	16	3/13	N/S	Spouse – 3
										Child – 11
										Friend – 2
Petyaeva^23^	UK	Interviews	15 (patient and family members	N/S	N/S	N/S	N/A	N/A	N/A	N/A
Whybrow^29^	UK	Focus groups and interviews	12	0/12	90.3	Joint pain	N/A	N/A	N/A	N/A

LBP: low back pain; F: females; M: males; N: number of; N/A: not assessed; N/S: not stated; UK: United Kingdom; USA: United States of America.

Eight studies gathered data from people with dementia.^23,25,29,30,45,50,53,54^ This consisted of the views of 145 people with dementia, collected through focus groups in two studies^29,50^ and interviews in six studies.^23,25,30,45,53,54^ Six studies provided basic characteristics data on their cohort with dementia.^25,29,30,45,53,54^ This is summarised in Table 1.

Nine studies gathered data from family members or informal caregivers of people with dementia.^3,24,25,30,45,50,53,54^ This was collected from 116 participants using interviews in six studies^24,25,30,45,53,55^ and focus groups in three studies.^3,50,54^ The relationship of these informal caregivers to individuals with dementia was reported in five studies^24,30,45,53,55^ and summarised in Table 1.

Most evidence derived from the perspective of formal care workers. Data were gathered from care home staff in 19 studies of 510 individuals.^3,23,27,29,30,32,36–38,41,42,45–50,52,56^ Data were collected using focus groups in three studies^3,48,50^ and by interviews in nine studies.^23,30,36,41,45,46,47,49,52^ Surveys with qualitative findings were presented in three studies^27,32,42^ whilst four used a combination of focus groups and interviews.^29,37,38,56^ The professional roles of these care home workers ranged from care assistants, registered nurses, physicians and care home managers. The frequency of these is presented in Table 2.

**Table 2.** Characteristics of included studies where the participants were formal caregivers and healthcare professionals who care for and manage people with dementia and pain.

Study	Country origin	Data collection method	Formal caregiver				Healthcare professional				
			N	Gender (male/female)	Mean age (years)	Role	N	Gender (male/female)	Mean age (years)	Role	Setting
Barry^30^	UK	Interviews	16	2/14	36.4	Registered nurses and carers	N/A	N/A	N/A	N/A	N/A
Barry^27^	UK	Survey	96	10/86	N/S	Home managers	N/A	N/A	N/A	N/A	N/A
Barry^28^	UK	Survey	N/A	N/A	N/A	N/A	182	74/108	36.8	Pharmacists	Community
Bullock^54^	UK	Interviews	N/A	N/A	N/A	N/A	14	5/9	N/A	GPs, psychiatrists	Community
Burns^32^	UK	Survey	32	5/27	41	Registered nurses	N/A	N/A	N/A	N/A	N/A
Chang^37^	Australia	Focus groups and interviews	13	0	0	Registered nurses and carers	31	0	0	GP, registered nurses, social worker, recreational therapist, doctor; occupational therapist; bereavement counsellor	Hospital and community
Cohen-Mansfield^49^	USA	Interviews	29	3/26	N/S	Registered nurses, home managers and carers	N/A	N/A	N/A	N/A	N/A
Corbett^3^	UK	Focus groups	12	N/S	N/S	Carers	N/A	N/A	N/A	N/A	N/A
Fry^34,35^	Australia	Focus groups	N/A	N/A	N/A	N/A	80	13/67	N/S	Emergency department nurses	Hospital
Gilmore-Bykovskyo^46^	USA	Interviews	13	N/S	N/S	Registered nurses	N/A	N/A	N/A	N/A	N/A
Graham^51^	Canada	Interviews	N/A	N/A	N/A	N/A	53	7/46	38.6	Registered nurse and medic	Hospital
Harmon^39^	Australia	Observations and interviews	N/A	N/A	N/A	N/A	21	N/S	45	Registered nurses	Hospital
Jennings^61^	Ireland	Survey	N/A	N/A	N/A	N/A	157	N/S	N/S	GPs	Community
Kaasalainen^38^	Canada	Focus groups and interviews	66	RN 96% female = 23/24	N/S	Medics, registered nurses and carers	N/A	N/A	N/A	GP = 9	N/A
				RN 97% female = 32/33				Physicians: 67% male = 6/9			
Karlsson^40^	Sweden	Focus groups	N/A	N/A	N/A	N/A	11	0/11	42–63	Nurses	Community
Karlsson^41^	Sweden	Interviews	23	2/21	25–65	Registered nurses and carers	N/A	N/A	N/A	N/A	N/A
Kovach^52^	Canada	Interviews	30	N/S	44	Registered nurses and carers	N/A	N/A	N/A	N/A	N/A
Krupic^43^	Sweden	Survey	24	7/17	N/S	Registered nurses and carers	N/A	N/A	N/A	N/A	N/A
Krupic^42^	Sweden	Survey	N/A	N/A	N/A	N/A	51	15/36	N/S	Registered nurse	Hospital
Lichtner^25^	UK	Observations and interviews	N/A	N/A	N/A	N/A	52	N/S	N/S	Registered nurse, care assistants, medics, pharmacists, physiotherapists, ward managers and clinical educators	Hospital
Liu^53^	China	Interviews and focus groups	49	2/48	N/S	Registered nurses and carers	N/A	N/A	N/A	N/A	N/A
Martin^50^	Canada	Focus groups	25	N/S	N/S	N/S	N/A	N/A	N/A	N/A	N/A
Mentes^45^	USA	Interviews	11	0/11	N/S	Carers	N/A	N/A	N/A	N/A	N/A
Minaya-Freire^62^	Spain	Focus groups and survey	N/A	N/A	N/A	N/A	10	0/10	37.6	Registered nurses	Hospital
Monroe^48^	USA	Focus groups	29	N/S	N/S	Care home nurses	N/A	N/A	N/A	N/A	N/A
Parkman^47^	USA	Interviews	6	1/5	54	Registered nurses and carers	N/A	N/A	N/A	N/A	N/A
Peisah^36^	Australia	Interviews	20	N/S	N/S	Registered nurses and carers	N/A	N/A	N/A	N/A	N/A
Petyaeva^23^	UK	Focus groups	7	N/S	N/S	Registered nurses and carers	N/A	N/A	N/A	N/A	N/A
Rodgers^33^	UK	Survey	N/A	N/A	N/A	N/A	37	N/S	N/S	Geriatricians	Hospital
Seffo^44^	Sweden	Focus groups and interviews	N/A	N/A	N/A	N/A	21	5/16	26–55	Nurses A&E	Hospital
Whybrow^29^	UK	Focus groups and interviews	22	1/21	49.5	Carers	2	N/S	N/S	GPs	N/A residential homes

A&E: accident and emergency; F: females; GP: general practitioner; M: males; N: number of; N/A: not assessed; N/S: not stated; RN: registered nurse; UK: United Kingdom; USA: United States of America.

Thirteen studies gathered data from a variety of healthcare professionals. This included nine studies of 325 healthcare professionals based in hospitals,^24,25,33-35,39,42,44,51^ four studies with 364 healthcare professionals working in community services,^28,40,54,57^ whilst collected data from 31 healthcare professionals working in either hospital or community settings. The professional roles of healthcare professionals are outlined in Table 2. In total, data were collected using focus groups in four studies,^34,35,40,44^ interviews in four^25,39,51,54^ and survey methods in four studies.^28,33,42,57^ Combined focus group and interview methods were used by Chang et al.,^37^ whilst focus groups and surveys were used together by Minaya-Feire et al.^24^

### Critical appraisal

A summary of the CASP critical appraisal results is presented in Supplementary File 2. The included studies presented with several recurrent limitations. Most frequently this related to not adequately considering the relationship between the researcher(s) and participants (*n* = 27; 82%), presenting insufficient evaluation on potential ethical issues which may have impacted on study robustness (*n* = 19; 58%), insufficient reporting of the data analysis methods to aid interpretation (*n* = 11; 33%). However, the studies demonstrated strengths in the generation of clear research aims and objectives (*n* = 33; 100%) and adopting the appropriate paradigm to study their research questions (*n* = 33; 100%). Thirty studies (91%) clearly stated the findings of their analyses.

### Meta-ethnography

A summary of themes and subthemes generated are presented in Supplementary File 3. Findings from the analysis, including GRADE-CERQual assessment, are presented in the Summary of Findings Table (Table 3). The seven themes identified are explored below:

**Table 3.** Summary of findings table.

Review finding	Studies contributing to the review finding	Assessment of methodological limitations	Assessment of relevance	Assessment of coherence	Assessment of adequacy	Overall CERQual assessment	Explanation of judgement
Uncertainty on assessment methods	21/33 studies^3, 24, 25, 29, 32, 34, 36, 38, 40–42, 45–51, 60, 62, 63^	Moderate methodological concerns (large uncertainty regarding the relationship between researcher and participants adequately considered; analysis and sampling details inadequate)	Minor concerns (all included studies were exploring issues related to pain for people with dementia although some concepts of the studies were related to non-cognitively impaired participants)	Moderate concerns (range of study settings reflecting the variation in experiences and stakeholders involved in the care of people with dementia)	Minor concerns about adequacy (22 studies contributed to this review finding providing moderately rich data overall)	Moderate confidence	Grading due to moderate concerns regarding methodological limitations, moderate concerns on relevance and coherence but mild concerns on adequacy
Familiarisation promotes action in pain management	9/33 studies^28, 34, 42, 46, 47, 49, 50, 52, 62^	Moderate methodological concerns (uncertainty regarding the relationship between researcher and participants adequately considered; analysis and sampling details inadequate)	Minor concerns (all included studies were exploring issues related to pain for people with dementia although some concepts of the studies were related to non-cognitively impaired participants)	Moderate concerns (range of study settings reflecting the variation in experiences and stakeholders involved in the care of people with dementia)	Moderate concerns (nine studies contributed to this review finding)	Moderate confidence	Grading due to moderate concerns regarding methodological limitations, moderate concerns on adequacy and coherence but minor concerns on relevance
Hierarchical pain management	19/33 studies^3, 25, 29, 32–34, 36, 38, 41, 42, 45, 46, 48–50, 54, 61–63^	Moderate methodological concerns (large uncertainty regarding the relationship between researcher and participants adequately considered; analysis and sampling details inadequate)	Minor concerns (all included studies were exploring issues related to pain for people with dementia although some concepts of the studies were related to non-cognitively impaired participants)	Moderate concerns (range of study settings reflecting the variation in experiences and stakeholders involved in the care of people with dementia)	Minor concerns about adequacy (19 studies contributed to this review finding providing moderately rich data overall)	Moderate confidence	Grading due to moderate concerns regarding methodological limitations, moderate concerns on relevance and coherence but mild concerns on adequacy
Tension over treatment options	17/33 studies^24, 27, 29, 32, 34–38, 41, 45, 46, 48, 50, 52, 53, 62^	Moderate methodological concerns (large uncertainty regarding the relationship between researcher and participants adequately considered; analysis and sampling details inadequate)	Minor concerns (all included studies were exploring issues related to pain for people with dementia although some concepts of the studies were related to non-cognitively impaired participants)	Moderate concerns (range of study settings reflecting the variation in experiences and stakeholders involved in the care of people with dementia)	Minor concerns about adequacy (17 studies contributed to this review finding providing moderately rich data overall)	Moderate confidence	Grading due to moderate concerns regarding methodological limitations, moderate concerns on relevance and coherence but mild concerns on adequacy
Inequality of pain management for people with dementia	6/33 studies^3, 17, 19, 36, 46, 47^	Moderate methodological concerns (not clear in all studies regarding the relationship between researcher and participants adequately considered; analysis and sampling details inadequate in two studies)	Minor concerns (all included studies were exploring issues related to pain for people with dementia although some concepts of the studies were related to non-cognitively impaired participants)	Moderate concerns (range of study settings reflecting the variation in experiences and stakeholders involved in the care of people with dementia)	Moderate concerns (six studies contributed to this review finding)	Moderate confidence	Grading due to moderate concerns regarding methodological limitations, minor concerns on relevance but moderate concerns on regarding adequacy and coherence
Failings in training and education for all individuals that support people with dementia and pain	18/26 studies^3, 27–30, 32, 35–38, 42, 45–47, 50, 52, 53, 62^	Moderate methodological concerns (large uncertainty regarding the relationship between researcher and participants adequately considered; analysis and sampling details inadequate)	Minor concerns (all included studies were exploring issues related to pain for people with dementia although some concepts of the studies were related to non-cognitively impaired participants)	Moderate concerns (range of study settings reflecting the variation in experiences and stakeholders involved in the care of people with dementia)	Minor concerns about adequacy (18 studies contributed to this review finding providing moderately rich data overall)	Moderate confidence	Grading due to moderate concerns regarding methodological limitations, moderate concerns on relevance and coherence but mild concerns on adequacy
Benefits in managing pain with people who have dementia	8/33 studies^8, 23, 24, 34, 42, 46, 49, 50, 52^	Moderate methodological concerns (uncertainty regarding the relationship between researcher and participants adequately considered; analysis and sampling details inadequate)	Minor concerns (all included studies were exploring issues related to pain for people with dementia although some concepts of the studies were related to non-cognitively impaired participants)	Moderate concerns (range of study settings reflecting the variation in experiences and stakeholders involved in the care of people with dementia)	Moderate concerns (eight studies contributed to this review finding)	Moderate confidence	Grading due to moderate concerns regarding methodological limitations, moderate concerns on adequacy and coherence but minor concerns on relevance

#### Theme 1: Uncertainty on assessment methods

There was moderate certainty evidence from 22 studies regarding uncertainty on using validated tools. This occurred internationally, in both care home, community and hospital settings. There was a consistent message that validated tools were infrequently used and perceived as ‘blunt’ or ‘insensitive’ in pain assessment for people with dementia. There was overwhelming evidence that the most widely used approach to detect pain in people with dementia, irrespective of setting, was observation of changes in behaviour. This was reported in 15 studies.^3,24,25,29,36,38,40–42,45,46,49-51,57^

Both the assessment and reporting of pain for people with dementia was universally acknowledged as challenging. Lichtner et al.,^26^ in their interviews of hospital healthcare professionals, highlighted this difficulty:

“…unless they’re able to confirm it, if I said, “Oh, is it sore?” and they said no, I’d put, “Appears to be in pain, but denies it when asked…”

(Lichtner et al.^26^; Page 7; Physiotherapist, H1)

Barriers to the use of validated pain scores centred around access, but also time.^24,25,29,32,38,47,54^ This was in community care, hospitals and care home settings. This was compounded by variation in patient presentation and the need to assess individuals over a period to detect a change in ‘normal’ behaviour compared to a ‘snap-shot’ assessment.^24,25,55^

#### Theme 2: Familiarisation promotes action in pain management

There was moderate certainty evidence from nine studies that familiarisation with the person, their preferences, routines and behaviours were key factors to being able to detect pain.^32–34,38,41,42,45,54,57^ This was acknowledged in care home staff,^32,41,45^ community healthcare^54,57^ and hospital settings.^34,42^ This familiarisation was also recognised as facilitating other causes of agitation which were not pain-related. Whilst this was considered time-consuming, it was seen as an important approach to personalising treatment and to avoid over-medicating people when pain was not the cause of distress. This trial-and-error approach was highlighted in a quote from Gilmore-Bynovskyi et al.^46^:

“You turn on the light, I might get ice cream, I might turn on the television, along with that I would give them a pain pill…if they’re restless or have a temp, maybe they’re having discomfort from a UTI. So it’s a matter of elimination….It is kind of a hit and miss”

(Gilmore-Bynovskyi et al.^46^; Page 134; care workers)

Where staff were not familiar with the individual, treatment delivery (particularly analgesia) may be delayed.^3,24,49^ To overcome this, encouraging staff members to work with fewer residents/patients to aid familiarisation and gaining trust rather than caring for many different people was seen as advantageous by care home workers and managers.^45^ Karlsson et al.^41^ also suggested that developing such a professional relationship also fosters an advocacy role.

#### Theme 3: Hierarchical pain management

There was moderate certainty evidence of a hierarchical approach to cascading information on pain detection. Care workers, particularly in care homes, but also in hospital settings, reported a process that because they engage in more personal care activities such as washing and toileting, they were better able to make early, timely detection of pain.^3,24,29,41,48,54,56,57^ This would be reported to senior colleagues. However, there is some reluctance to do so regarding carer’s perceived qualification in some instances. Whybrow et al.^29^ highlighted this in a carer quote:

“You’ve got to be careful not to give an opinion. You’re not qualified, so you can’t give an opinion”

(Whybrow et al.^29^; Page 86; paid care, FG2)

This challenge was reported in four studies undertaken in care home, community and hospital setting.^3,25,54,56^

Even when pain is detected, there was a reluctance to foster and use connections to evaluate treatment outcomes. Barry et al.^30^ and Corbett et al.^3^ reported pain the person with dementia in their family experienced was undertreated and unnoticed by care home staff. They reported that family members wished for greater involvement in pain management. Irrespective of the type of treatment, there was a reluctance to use any form of post-treatment evaluation across the literature, be that acute hospital care or home-based care.^3,36,46,56,57^

#### Theme 4: Tension over treatment options

There was a consistent theme from moderate certainty evidence that carers and healthcare workers had a reluctance to suggest non-pharmacological methods of pain relief which they could offer, feeling they did not have the training to offer such approaches. There was evidence of perceived under-utilisation of non-pharmacological interventions by healthcare professionals. Whilst there was moderate certainty evidence that many carers and healthcare professionals believe non-pharmacological treatments can be helpful in managing pain for people with dementia,^27,36,45,46,50,54^ there was far greater uncertainty, variability and conflict in support for analgesia. This inconsistency was also shared amongst people with dementia.^54^ This was illustrated in the quote from an individual with dementia living in the community

“I hate taking tablets at the best of times, so I’ve got to be getting pretty bad before I’ll take them…I’ve got an aversion to taking poisons…Every tablet is a poison of some kind”

(Bullock et al.^54^; Page 9; Person with Dementia)

There were repeated fears, particularly from family members and informal caregivers, care staff and some healthcare professionals on the side-effects of analgesics.^27,32,35–37,46,52^ Particular concerns included over-medication, sedation, associated falls and complications from multi-morbidity, particularly gastric and cognitive complications. There was agreement in the views of family members and healthcare professionals that when there was a clearer indication for pain, that is, post-surgical or trauma, the use of medications was considered justified.^25,34,35,42^ There was greater uncertainty when there was no clear pathology or trauma.^27,38,41,50^ This was particularly evident in the responses from care home workers where uncertainty existed over whether pain was a consequence of ageing and whether people with dementia perceive pain equally to those without dementia.^50^ This was at odds with family members where there was a balance between those who do or do not wish for their friends and family members to receive medication.^24,27,29,34,45,48,54^ The balance between over- and under-medicating individuals to ensure pain relief without the harmful physical or social disadvantages which this may pose was evident. Education and communication across all involved was deemed as key to overcome this.

#### Theme 5: Inequality of pain management for people with dementia

There was moderate certainty evidence from six studies indicating inequality of pain management for people with dementia. Three studies highlighted different approaches offered to people with dementia depending on their presenting health. For instance, individuals who were admitted to hospital medical wards were considered to receive poorer pain management than those on surgical wards.^3,25,46^ Whilst this was in-part associated with the expectation of pain post-operatively,^3,25^ there were notable structural and educational differences in using validated pain assessments and doctor and senior nurse perspectives on the importance of pain management across medical versus surgical specialities.^25,46^ This organisational viewpoint was mirrored in the care home setting where, in three studies,^3,27,47^ care home workers felt that variation in pain management was a function of leadership priorities. In this, pain management was considered better when regarded as having greater importance by institutional leads. Peisah et al.^36^ recommended that one approach to overcoming variation may be through national standards to monitor performance in care homes on pain management approaches. They cited the UK’S Care Quality Commission as one regulator who may mandate improvements in pain management for people with dementia living in care homes.

#### Theme 6: Failings in training and education for all individuals that support people with dementia and pain

Many respondents had a low opinion of the potential effectiveness of pain management for people with dementia. This was associated with poor training and understanding on how pain ‘should’ be managed. There was moderate certainty evidence from three studies indicating that whilst people supporting individuals with dementia feel they are not adopting evidence-based treatment approaches,^28,36,38^ what is adopted is frequently a patient-focused, perceived trial-and-error approach.^27,28,36,37,46,52^ This was particularly highlighted in studies from care home staff members where incremental changes to support could be made to determine which approach helped, that is, repositioning, personal care, distraction and medication. There was acknowledgement that pain management approaches should be taught to family members and friends of care home residents who want to ensure their friends/family are not in discomfort. Both Fry et al.^34^ and Barry et al.^30^ from hospital and care home settings, respectively, acknowledged a disconnect between family members and healthcare professionals particularly on medication for pain relief. Overcoming this disconnect was seen as important. Fry et al.^34^ highlighted the ability of family members and caregivers to be able to help patients admitted to hospital in reducing agitation. One direct quote to illustrate this was:

“They don’t really know why they are in hospital or that they have a broken bone, but seeing a familiar family member just makes them settle, relaxed and comfortable”

(Fry et al.^34^; Page 1327; G15)

Whilst education was considered important across settings, experience was also perceived as important^3,29,35^ where more experienced and knowledgeable staff were considered to offer better pain management. This chimes with the processes of cascading information from carers or family members to more experienced members to make decisions on management options, but also acknowledging the value of experiential learning both on pain management *per se* but also supporting individuals and their personal needs. Such notions re-enforced the familiarisation concept with perceived ‘better’ pain management offered to those who are familiar to the decision-makers.

#### Theme 7: Benefits in managing pain with people who have dementia

There was moderate certainty evidence from across settings from eight studies^23,24,34,42,46,49,50,52^ that managing pain offers multiple benefits to people with dementia particularly in reducing agitation and increasing quality of life with supporting people. This is highlighted in the quote from Petyaeva et al.^23^:

“…we decided to change the tablets into liquid medication and now she’s taking it regularly. All of a sudden, she’s going to music therapy. She’s going to activities. She’s eating like never – she’s like a totally different person”

(Petyaeva et al.^23^; Page 226; FG1)

As Fry et al.^34^ acknowledge, these people have an ethical right to good pain management. There were also associated benefits acknowledged by three studies of improved pain providing caregivers with reduced burden either in hospital^34^ or care home settings.^47,52^ Furthermore, successful pain management offered both caregivers and staff the opportunity to engage in more social activities which offer both health and wellbeing benefits for the person with dementia and the people around them.^34,50^

#### Line of argument

**Figure 2.**
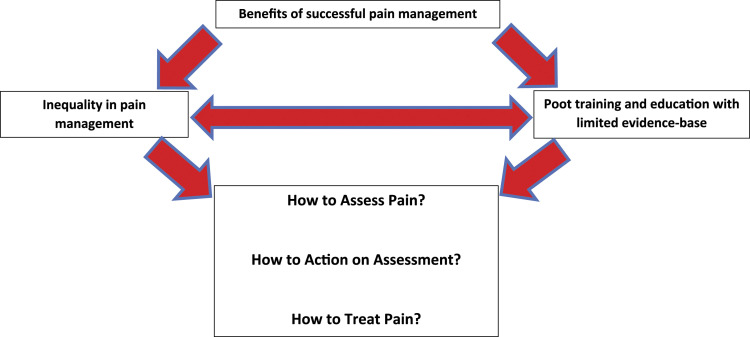
Schema of line of argument.

The line of argument developed from this analysis is presented as a scheme as Figure 2. This illustrates that inequality of care from various drivers, and low training and evidence influence the assessment processes, actions and treatment offered to people with dementia and pain from those who support them. As illustrated, understanding potential benefits of offering ‘good’ pain management to these people may influence the motivation on addressing inequality and training/research agendas to improve the care offered to these individuals.

## Discussion

The findings of this study highlight challenges faced in managing pain for people with dementia and those who support them. Pain is an individual experience. Understanding the ‘usual’ behaviours and habits of a person with dementia, be that by family members or friends, care home workers or healthcare professionals, is paramount. The overlapping experiences of other sources of distress and agitation make the assessment and management of pain in people with dementia unique. This is compounded by the uncertainty over how medications should be used, which is, in the main, unlike the experiences of people without dementia. Given these complexities, individuals caring for people who have dementia feel under-prepared and under-served with training and research to inform care, a sentiment which is echoed by family members and friends of people with dementia. Where successfully managed, there is a clear focus on pain management as a priority, with appropriate communication, re-evaluation and training of staff to support these individuals. Through such approaches, these people can have good pain management strategies which benefits not only on their overall health and wellbeing but also reduces stress and anxiety on friends, families and carers who support them.

There appeared a uniform perspective of the difficulties of pain management in hospital, care home and community settings. This was consistent globally and reflects the paucity of guidelines regarding the assessment and management of pain for people with dementia.^54^ Given variation in how community and social care is structured in countries such as the UK, Scandinavia, Australia and USA, this was surprising. For instance, whilst pain management is rarely mandated through policy recommendations in long-term care facilities, the Canadian provinces of Alberta, Saskatchewan and Ontario have mandated the use of the interRAI suite of assessment tools for assessing and reporting pain.^55^ Nonetheless, this homogeneity of perspectives reinforces this as a major health challenge. Consideration on approaches to improve care to strengthen the management for people with dementia and pain may require more local-level perspectives. Nonetheless, consideration on how this should be adopted, particularly considering education and more focused, stepwise treatment pathways, should be considered. Manietta et al.^56^ recently reported the outcomes of algorithm-based or protocolised pain management approaches for people with dementia living in nursing homes. They reported no clear benefit of these approaches compared to pain education. They recommended further research on evidence-based pain management strategies. The findings of our work re-enforce this, highlighting that both nursing homes, healthcare professionals and caregivers remain uncertain on how best to support people with dementia who experience pain.

There was a consistent message regarding caregiver involvement for the support of people with dementia who experience pain. Studies such as Bullock et al.^24^ and Corbett et al.^3^ highlight a mismatch between the understanding of family members to pain severity experienced by the person with dementia versus those suggested by care home or hospital teams. This can be a source of anxiety, particularly for family members.^30,34^ Bullock et al.^24^ highlighted the value of engaging family members in the assessment and management of pain for people with dementia. This can include in history-taking, adherence to medication, advocacy to healthcare professionals and familiarisation which they offer to people with dementia to both reduce agitation and distract the individual with pain. Riffin et al.^57^ reported that caregivers wish to improve the support of their friends and family members who have dementia and pain, but their emotions, the communication challenges and uncertainties around what best to do, frequently make collaborating with healthcare professionals inadequate from their perspectives. Consideration on how care home workers and healthcare professionals educate family members around pain management for people with dementia may be equally important as providing greater education to formal caregivers and should be a research consideration for the future.

This study has identified a wealth of evidence regarding the perspectives and experiences of individuals who support people with dementia who have pain. There is evidence from across the patient pathways from community and care home services to hospital care. However, there is a need to better understand specifically the experiences of people who have dementia. This is a major limitation in the literature. Better understanding the views of those who are at the centre of this experience is critically important, with limited presented in the evidence. This should be both from the developing and designing of research studies, in addition to the formal exploration of the views and experiences of people with dementia living with pain. Secondly, the evidence base has focused on care home and hospital settings. Limited evidence exists regarding individuals with dementia who live in the community, in their own homes. This is important given key differences between people living in the community compared to long-term care facilities. For example, there are differences in the level of self-reported pain assessment which can be offered in those with mild or moderate cognitive impairment,^58^ involvement of informal caregivers^6^ and access to healthcare services which differs between community settings compared to long-term care facilities.^59^ Exploration of how pain impacts on their lives, and the lives of people they may live with who also may have pain, would therefore be valuable. Finally, there was limited evidence characterising the religious, ethnic or social backgrounds of people with dementia who have pain. Given this study has highlighted the individualisation of pain, its meaning and how it should be managed through a person-centred approach, further consideration on these contextual factors should be explored.

This study presented with several strengths and limitations. Two key strengths included the global approach to the evidence and exploring the perspectives of all major ‘players’, from people with dementia, their friends and family, care home staff and healthcare professionals. This overarching cross-setting approach meant that the complex interactions in pain management were considered. Several key limitations should be considered. Firstly, the focus of this study was on people with dementia. Therefore, people with other forms of cognitive impairment, most notably delirium, were not considered. This was deemed prudent given its transient nature. Nonetheless, this was not explored in this study. Secondly, the included studies poorly explored potential differences in outcomes by stage or severity of dementia. Whilst there was a focus on people with more severe cognitive impairment, it was not possible to formally assess this as the reporting of stage of dementia was poor across the literature. This is a recommended area for future study. Finally, the interpretation of the findings was based on team discussion across the review team. However, we did not include experts by experience, that is, patient or caregiver viewpoints. This was deemed appropriate as we wished the evidence to provide the findings, rather than be interpreted by personal perspectives to influence third-order interpretation. Nonetheless, if the findings of this study were taken forward to consider clinical implementation, further consultation and stakeholder involvement with such individuals would be advisable.

## Conclusion

The findings of this meta-ethnography highlight the challenges faced by people with dementia who have pain and those who support them. The detection and subsequent management of pain is confusing for all but experts. This is a concern given the expected increased prevalence of dementia within an ageing population. Improvements in education for individuals who care for these people would be valuable across health and social care pathways. Furthermore, supporting family members and relatives on pain experiences and treatment options could improve awareness to reduce their own anxieties, which are often reported. Such a global, holistic approach could improve the experiences of all involved in managing pain.

## Supplemental Material

Supplemental Material - Pain management for people with dementia: a cross-setting systematic review and meta-ethnography Click here for additional data file. Supplemental Material for Pain management for people with dementia: a cross-setting systematic review and meta-ethnography by To Smith, Dawn Lockey, Helen Johnson, Lauren Rice, Jay Heard and Lisa Irving in British Journal of Pain
