# Supplementary material for: Pain management for people with dementia: a cross-setting systematic review and meta-ethnography
Source: Br J Pain. 2022 Sep 16;17(1):6–22. doi: 10.1177/20494637221119588 (PMC9940246; doi:10.1177/20494637221119588)
Supplement: Supplemental Material - Pain management for people with dementia: a cross-setting systematic review and meta-ethnography [file sj-pdf-1-bjp-10.1177_20494637221119588.pdf]

Supplementary File 1: Search strategy (EMBASE example – optimised for other databases)

1. exp Pain/
2. exp Pain Threshold/
3. exp Pain Perception/
4. exp Myalgia/
5. exp Neuralgia/
6. exp Acute Pain/
7. exp Chronic Pain/
8. exp Hyperalgesia/
9. exp Neuritis/
10. exp Paresthesia/
11. (pain or discomfort or allodynia, or neuritis or neuropathy or myalgia or neuralgia or hyperalgesia or paresthesia or soreness or ache* or dys?sthesia or Nocicepti*).ti,ab
12. OR/1-11
13. (aged or elder* or seniors or (old* adj2 (people or person* or patient* or men or women))).mp.
14. exp dementia/
15. exp Alzheimer Disease/
16. exp Cognition Disorders/
17. cognitive impairment.mp.
18. Cognitive function*.mp.
19. (alzheimer* or dement* or "Frontotemporal lobar degeneration" or "Frontotemporal dement*" or Huntington or "Lewy Body disease").tw.
20. OR/14-19
21. AND/13,20
22. exp research, qualitative/
23. exp attitude to health/
24. exp interviews as topic/
25. exp focus groups/
26. exp life experiences/
27. Interview.ti,ab
28. (focus group).ti,ab
29. Views.ti,ab
30. Perspectives.ti,ab
31. (live* adj2 experience).ti,ab
32. Attitudes.ti,ab
33. Opinions.ti,ab
34. Experiences.ti,ab
35. (qualitative OR ethno* OR emic OR etic OR phenomenolog).ti,ab
36. (focus AND group* OR grounded AND theory OR narrative AND analysis OR lived AND experience* OR life).ti,ab
37. (theoretical AND sampl* OR purposive AND sampl* OR ricoeur OR spiegelberg* OR merleau).ti,ab
38. (field AND note* OR field AND record* OR fieldnote* OR field AND stud*).ti,ab
39. (participant* adj3 observ*).ti,ab
40. (unstructured AND categor* OR structured AND categor*).ti,ab
41. OR/22-40
42. AND/21,41

Supplementary File 2: Summary of the CASP critical appraisal results

Study	CASP Qualitative Criterion									
	1	2	3	4	5	6	7	8	9	10
Barry [30]	Y	Y	N	Y	N	N	N	N	Y	N
Barry [28]	Y	Y	N	Y	N	N	N	Y	Y	N
Barry [27]	Y	Y	N	Y	N	N	N	N	Y	N
Burns [32]	Y	Y	N	Y	N	N	N	N	Y	Y
Bullock [57]	Y	Y	Y	Y	Y	N	Y	Y	Y	Y
Chang [37]	Y	Y	Y	Y	Y	Y	Y	Y	Y	Y
Cohen-Mansfield [49]	Y	Y	N	Y	N	N	N	N	Y	Y
Corbett [3]	Y	Y	Y	Y	Y	N	Y	Y	Y	Y
Fry [35]	Y	Y	Y	Y	Y	N	Y	Y	Y	Y
Fry [36]	Y	Y	Y	Y	Y	N	Y	Y	Y	Y
Gilmore-Bykovskyo [46]	Y	Y	N	N	N	N	N	Y	Y	N
Graham [51]	Y	Y	N	N	Y	N	Y	N	Y	N
Harmon [39]	Y	Y	Y	Y	Y	Y	Y	Y	Y	Y
Jennings [62]	Y	Y	N	N	N	N	Y	N	Y	N
Kaasalainen [38]	Y	Y	Y	Y	Y	Y	N	Y	Y	Y
Karlsson [41]	Y	Y	Y	Y	Y	Y	Y	Y	Y	Y
Karlsson [40]	Y	Y	Y	Y	Y	N	Y	Y	Y	N
Kovach [52]	Y	Y	Y	Y	Y	N	N	Y	Y	Y
Krupic [43]	Y	Y	N	Y	N	N	N	N	Y	N
Krupic [42]	Y	Y	N	Y	N	N	N	N	Y	N
Lichtner [25]	Y	Y	Y	Y	Y	Y	Y	Y	Y	Y
Lichtner [26]	Y	Y	Y	Y	Y	Y	Y	Y	Y	Y
Liu [64]	Y	Y	Y	Y	Y	N	N	Y	Y	Y
Liu [53]	Y	Y	Y	Y	Y	N	N	Y	N	Y
Malhortra [61]	Y	Y	Y	Y	Y	N	Y	Y	Y	Y
Martin [50]	Y	Y	Y	Y	Y	N	N	Y	Y	Y
Mentes [45]	Y	Y	Y	Y	Y	N	N	Y	Y	Y
Minaya-Freire [63]	Y	Y	Y	Y	Y	N	N	Y	Y	Y
Monroe [48]	Y	Y	Y	Y	Y	N	Y	Y	Y	N
Parkman [47]	Y	Y	Y	N	Y	Y	N	Y	N	N
Peisah [36]	Y	Y	Y	Y	Y	N	Y	N	Y	Y
Petyaeva [23]	Y	Y	N	N	Y	N	Y	N	N	N
Rodgers [33]	Y	Y	N	N	N	N	N	N	Y	Y
Seffo [44]	Y	Y	Y	Y	N	N	N	Y	Y	N
Whybrow [29]	Y	Y	Y	Y	Y	N	N	Y	Y	Y

Y = Satisfied; N = Not satisfied

CASP Critical Appraisal Criteria:

1. Was there a clear statement of the aims of the research?
2. Is a qualitative methodology appropriate?
3. Was the research design appropriate to address the aims of the research?
4. Was the recruitment strategy appropriate to the aims of the research?
5. Were the data collected in a way that addressed the research issue?
6. Has the relationship between researcher and participants been adequately considered?
7. Have ethical issues been taken into consideration?
8. Was the data analysis sufficiently rigorous?
9. Is there a clear statement of findings?
10. Is the research valuable to clinical practice?

Supplementary File 3: Summary of themes and subthemes generated from the analysis

**Uncertainty on assessment methods**	**Studies**
Behaviour of Person with Dementia used most frequently	Peisah 2004; Graham 2020; Karlsson 2012; Gilmore-Bykovyo 2013; Krupic 2018; Lichtner 2016; Corbett 2016; Maertin 2005; Karlsson 2015; Monroe 2015; Mentes 2004; Whybrow 2018; Jenning 2018; Minaya-Freire 2020; Cohen-Mansfield 2002
Challenging to use pain scores due to time and access	Kaasalainen 2007; Burns 2015; Bullock 2020; Lichtner 2016; Parkman 2021; Whybrow 2018; Minaya-Freire 2020
Challenging using pain scores due to inconsistency of patient presentation	Malhortra 2021; Bullock 2020; Lichtner 2016
Challenge relying on 3rd person who are not available to assist in corroborating pain score	Fry 2015; Bullock 2020; Lichtner 2016; Minaya-Freire 2020

**Familiarisation promotes action in pain management**	**Studies**
Caregivers and care assistants and family key in determining if patient is 'normal of them' – familiarisation of patient is key for this	Rodgers 2015; Kaasalainen 2007; Burns 2015; Fry 2015; Bullock 2020; Krupic 2018; Karlsson 2015; Mentes 2004; Jennings 2018
Familiarisation is around understanding the person with dementia and them normally. These are often the advocates and take on the role. If not familiar to the Person with Dementia then getting analgesia and management takes longer	Gilmore-Bykovyo 2013; Liu 2014; Corbett 2016; Karlsson 2015; Mentes 2004; Minaya-Freire 2020; Cohen-Mansfield 2002

**Hierarchical pain management**	**Studies**
To get medication, the nurse is the gate-keeper and relies on caregivers/care assistants and family to say if the patient isn't 'normal for them' – clear hierarchy.	Liu 2014; Bullock 2020; Lichtner 2016; Corbett 2016
There is a barrier between nursing teams and GPs and wider AHP on accessing medications particularly with reluctance from those who don't know the patient as well.	Peisah 2004; Kaasalainen 2007; Burns 2015; Liu 2014; Martin 2005; Whybrow 2018; Minaya-Freire 2020
There is limited re-evaluation of the cascade of information is for the detection of pain not from any form of evaluation of effectiveness of treatment.	Peisah 2004; Gilmore-Bykovyo 2013; Liu 2014; Corbett 2016; Jennings 2018
Carers in roles managing more personal care in strong position to be able to cascade information to others.	Liu 2014; Bullock 2020; Corbett 2016; Karlsson 2015; Monroe 2015; Whybrow 2018; Jennings 2018; Minaya-Freire 2020

**Tension over treatment options**	**Studies**
Medication seen as last resort for pain management, once non-medication approaches have failed.	Gilmore-Bykovyo 2013; Bullock 2020; Martin 2005; Barry 2012
Challenges over providing medication when there is uncertainty over diagnosis and pain levels with inaccuracy of measure (perceived).	Kaasalainen 2007; Martin 2005; Karlsson 2015; Barry 2012
Fear over side effects from all individuals.	Peisah 2004; Chang 2019; Burns 2015; Fry 2016; Gilmore-Bykovyo 2013: Barry 2012; Kovach 2000
Uncertainty over whether pain is normal in ageing.	Martin 2005
Uncertainty over whether Person with Dementia feel pain	Martin 2005

in the same was as people without dementia.	
Tension between family members and carers on whether to medicate or not (over vs. under) and registered nurses or physicians.	Fry 2015; Bullock 2020; Barry 2012; Monroe 2015; Mentes 2004; Whybrow 2018; Minaya-Freire 2020
Belief that other diseases take priority and pain doesn't get the attention it needs.	Liu 2018; Whybrow 2018;

**Inequality of pain management for people with dementia**	**Studies**
Pain management differs for different patient groups i.e. medical vs surgical.	Gilmore-Bykovyo 2013; Lichtner 2016; Corbett 2016
Variation in care homes with some have good pain management leadership and others don't.	Parkman 2021; Barry 2012
Requirement for a CQC or national standard.	Peisah 2004

**Failings in training and education for all individuals that support people with dementia and pain**	**Studies**
Overcoming misconceptions on pain, ageing and dementia required.	Burns 2015; Liu 2018; Martin 2005
There is limited use of evidence-based approaches to pain management.	Peisah 2004; Kaasalainen 2007; Barry 2013
More experienced and knowledgeable nurses provide better care.	Fry 2016; Corbett 2016; Whybrow 2018
There is an acceptance on a trial-and-error approach to pain management rather than a step-wise approach.	Peisah 2004; Chang 2019; Gilmore-Bykovyo 2013; Barry 2012; Barry 2013; Kovach 2000
Family member training is also needed as they perceive the person is in greater pain than they are. Expectation matching may be needed.	Fry 2015; Barry 2015
Rare to get a diagnosis of pain or source of pain once someone enters a care home.	Barry 2015; Whybrow 2018
Pain is not regarded a vital sign and therefore has lower priority – training and understanding is considered a reason for this.	Liu 2018; Parkman 2021; Krupic 2018; Corbett 2016; Whybrow 2018; Minaya-Freire 2020
Uncertain on key information i.e. not known about specific pain assessment tools or how to manage. Uncertain on whether pain should be managed differently for a Person with Dementia vs. Person Without Dementia.	Burns 2015
Perceived underestimation of benefit of non-pharmacological treatment from nurses.	Peisah 2004; Mentes 2004

**Benefits in managing pain with people who have dementia**	**Studies**
Pain management can also manage aggressive symptoms.	Fry 2015; Gilmore-Bykovyo 2013; Krupic 2018; Martin 2005; Barry 2013; Minaya-Freire 2020; Kovach 2000; Cohen-Mansfield 2002
People with Dementia have an ethical right.	Fry 2015
Reduces carer burden as makes caring for a Person with Dementia easier if they are more comfortable.	Fry 2015; Parkman 2021; Kovach 2000
Reducing pain means individuals can engage in more social activities for cognitive stimuli. Added health and wellbeing benefit.	Fry 2015; Martin 2005
However – do some People with Dementia use it to get attention.	Cohen-Mansfield 2002

Supplementary File 4: PRISMA Checklist

Section/topic	#	Checklist item	Reported on page #
**TITLE**			
Title	1	Identify the report as a systematic review, meta-analysis, or both.	Title
**ABSTRACT**			
Structured summary	2	Provide a structured summary including, as applicable: background; objectives; data sources; study eligibility criteria, participants, and interventions; study appraisal and synthesis methods; results; limitations; conclusions and implications of key findings; systematic review registration number.	Abstract
**INTRODUCTION**			
Rationale	3	Describe the rationale for the review in the context of what is already known.	Intro Para 1&3
Objectives	4	Provide an explicit statement of questions being addressed with reference to participants, interventions, comparisons, outcomes, and study design (PICOS).	Intro Para 4
**METHODS**			
Protocol and registration	5	Indicate if a review protocol exists, if and where it can be accessed (e.g., Web address), and, if available, provide registration information including registration number.	Methods Para 1
Eligibility criteria	6	Specify study characteristics (e.g., PICOS, length of follow-up) and report characteristics (e.g., years considered, language, publication status) used as criteria for eligibility, giving rationale.	Methods; Eligibility Criteria
Information sources	7	Describe all information sources (e.g., databases with dates of coverage, contact with study authors to identify additional studies) in the search and date last searched.	Methods; Search Strategy
Search	8	Present full electronic search strategy for at least one database, including any limits used, such that it could be repeated.	Supplement Table 1
Study selection	9	State the process for selecting studies (i.e., screening, eligibility, included in systematic review, and, if applicable, included in the meta-analysis).	Methods; Eligibility Criteria

Data collection process	10	Describe method of data extraction from reports (e.g., piloted forms, independently, in duplicate) and any processes for obtaining and confirming data from investigators.	Methods; Data Extraction
Data items	11	List and define all variables for which data were sought (e.g., PICOS, funding sources) and any assumptions and simplifications made.	Methods; Data Extraction
Risk of bias in individual studies	12	Describe methods used for assessing risk of bias of individual studies (including specification of whether this was done at the study or outcome level), and how this information is to be used in any data synthesis.	Methods; Methodological Quality Assessment
Summary measures	13	State the principal summary measures (e.g., risk ratio, difference in means).	Methods; Data Synthesis
Synthesis of results	14	Describe the methods of handling data and combining results of studies, if done, including measures of consistency (e.g., I^2) for each meta-analysis.	Not Applicable

Section/topic	#	Checklist item	Reported on page #
Risk of bias across studies	15	Specify any assessment of risk of bias that may affect the cumulative evidence (e.g., publication bias, selective reporting within studies).	Methods Quality Assessment and Assessment of GRADE-CERQual
Additional analyses	16	Describe methods of additional analyses (e.g., sensitivity or subgroup analyses, meta-regression), if done, indicating which were pre-specified.	Methods Data Synthesis
**RESULTS**			
Study selection	17	Give numbers of studies screened, assessed for eligibility, and included in the review, with reasons for exclusions at each stage, ideally with a flow diagram.	Results Search Results & Figure 1
Study characteristics	18	For each study, present characteristics for which data were extracted (e.g., study size, PICOS, follow-up period) and provide the citations.	Results; Characteristics of Studies &

			Table 1
Risk of bias within studies	19	Present data on risk of bias of each study and, if available, any outcome level assessment (see item 12).	Results; Critical Appraisal
Results of individual studies	20	For all outcomes considered (benefits or harms), present, for each study: (a) simple summary data for each intervention group (b) effect estimates and confidence intervals, ideally with a forest plot.	Results, Meta-Ethnography
Synthesis of results	21	Present results of each meta-analysis done, including confidence intervals and measures of consistency.	Not Applicable
Risk of bias across studies	22	Present results of any assessment of risk of bias across studies (see Item 15).	Results; Critical Appraisal & Supplementary File 2
Additional analysis	23	Give results of additional analyses, if done (e.g., sensitivity or subgroup analyses, meta-regression [see Item 16]).	Results: Meta-Ethnography
**DISCUSSION**			
Summary of evidence	24	Summarize the main findings including the strength of evidence for each main outcome; consider their relevance to key groups (e.g., healthcare providers, users, and policy makers).	Discussion Para 1
Limitations	25	Discuss limitations at study and outcome level (e.g., risk of bias), and at review-level (e.g., incomplete retrieval of identified research, reporting bias).	Discussion Para 5
Conclusions	26	Provide a general interpretation of the results in the context of other evidence, and implications for future research.	Discussion Para 2-4
**FUNDING**			
Funding	27	Describe sources of funding for the systematic review and other support (e.g., supply of data); role of funders for the systematic review.	Declarations

From: Moher D, Liberati A, Tetzlaff J, Altman DG, The PRISMA Group (2009). Preferred Reporting Items for Systematic Reviews and Meta-Analyses: The PRISMA Statement. PLoS Med 6(6): e1000097. doi:10.1371/journal.pmed1000097

For more information, visit: **www.prisma-statement.org**.
